# Nanoemulsions and Microemulsions for Intranasal Drug Delivery: A Bibliometric Analysis and Emerging Trends (2004–2024)

**DOI:** 10.3390/pharmaceutics17091104

**Published:** 2025-08-25

**Authors:** Ariane Krause Padilha Lorenzett, Vanderlei Aparecido de Lima, Clovis Orlando Pereira da Fonseca, Rubiana Mara Mainardes

**Affiliations:** 1 Laboratory of Nanostructured Formulations, Universidade Estadual do Centro-Oeste, Alameda Élio Antonio Dalla Vecchia St., 838, Guarapuava 85040-167, PR, Brazil; arianekrausepad@gmail.com; 2 Chemistry Department, Federal Technological University of Paraná UTFPR, Via do Conhecimento, s/n, KM 01, Fraron, Pato Branco 85503-390, PR, Brazil; valima@utfpr.edu.br; 3 Department of Neurological Surgery, Federal Hospital of Ipanema, Rio de Janeiro 22411-020, RJ, Brazil; clovis.orlando@uol.com.br

**Keywords:** scientometry, nanoemulsion, microemulsion, intranasal, Bibliometrix

## Abstract

**Background/Objectives**: Nanoemulsions and microemulsions are promising drug delivery systems capable of enhancing the solubility, stability, and bioavailability of active pharmaceutical ingredients, particularly for central nervous system (CNS) disorders. This study presents a bibliometric analysis of scientific publications on intranasal nanoemulsions from 2004 to 2024, based on data from the Scopus database. **Methods**: A total of 379 articles were analyzed using Bibliometrix and VOSviewer to identify publication trends, leading countries and institutions, prominent journals, and keyword networks. **Results:** Publications grew significantly over the last decade, with India, the United States, and China leading in volume. Keyword analysis revealed strong thematic clusters related to “brain targeting,” “drug delivery,” and “intranasal administration,” highlighting this route’s potential for bypassing the blood–brain barrier. The most studied compounds included curcumin, quercetin, carbamazepine, diazepam, and insulin, each with therapeutic applications in neurodegenerative and psychiatric disorders. **Conclusions:** The findings highlight growing interest in intranasal nano- and microemulsions as a non-invasive and efficient CNS delivery strategy. Future research can bridge translational gaps, enhancing efficacy and safety while meeting regulatory expectations for patient-centered drug development. This study provides a comprehensive overview of current trends and serves as a guide for advancing innovative intranasal delivery platforms.

## 1. Introduction

Nanoemulsions are colloidal systems composed of oil-in-water (O/W) or water-in-oil (W/O) emulsions, in which the dispersed droplets range in size within the nanometric scale (typically between 20 and 200 nm) [1]. These formulations are produced using high-energy techniques, such as high-pressure homogenization or ultrasonication. Owing to the presence of surfactants and the small droplet size, nanoemulsions exhibit good kinetic stability, preventing phenomena such as droplet coalescence, aggregation, or sedimentation [1,2]. In the pharmaceutical field, nanoemulsions have gained prominence due to their ability to enhance the absorption of poorly soluble drugs, protect active compounds from chemical and enzymatic degradation, and facilitate tissue penetration and the crossing of physiological barriers, such as the nasal mucosa and, in certain cases, the blood–brain barrier [2,3,4].

Microemulsions, in turn, although presenting a similar composition, are fundamentally distinguished by their thermodynamic stability [5]. These systems are homogeneous and transparent, with droplet sizes smaller than 100 nm, formed spontaneously through the mixing of oil, water, surfactants, and co-surfactants, resulting in a structure characterized by negative interfacial energy [6,7,8]. This characteristic enables the simultaneous solubilization of both hydrophilic and lipophilic compounds, thereby expanding their applicability across various routes of administration, including oral, topical, ophthalmic, injectable, and intranasal delivery [9,10]. Despite their versatility, the high surfactant content may pose toxicological risks, particularly when applied to mucosal surfaces, thereby requiring careful evaluation of the system’s safety [5,11].

Although microemulsions and nanoemulsions are fundamentally distinct systems, their terminology is frequently misused or confused in the scientific literature due to the similarity of the prefixes “micro” and “nano.” Microemulsions were initially defined as thermodynamically stable systems that form spontaneously, typically without the need for high-energy input, and often with droplet sizes below 100 nanometers [6,7,8]. In contrast, nanoemulsions are kinetically stable systems that generally require high-energy methods, such as sonication or high-pressure homogenization, for their formation, and typically exhibit droplet sizes ranging from 20 to 200 nanometers [1]. However, an important characteristic of nanoemulsions is their smaller droplet size range, between 1 and 100 nm, which imparts unique properties not observed in standard micellar solutions. Owing to this size distribution, they allow light to pass through without distortion or refraction, giving them a translucent appearance. They can be prepared either by using high-energy instruments or by employing a microemulsion as a template for nanoemulsion formation. Although nanoemulsions are not as thermodynamically stable as microemulsions, they can nevertheless remain preserved for decades [12]. While the terms “nano” and “micro” are clearly differentiated in the fields of chemistry and physics—referring to structures below and above 1000 nm, respectively. This distinction is often less rigorously applied in pharmaceutical sciences. As a result, it is common to find studies that inaccurately label nanoemulsions as microemulsions, and vice versa [13,14]. This inconsistency has led to the misclassification and mis-indexing in numerous publications, contributing to ongoing confusion in the field.

The intranasal route has gained prominence as an alternative and non-invasive method for drug administration, being particularly advantageous for compounds with poor oral absorption or those requiring rapid onset of action [15]. The nasal mucosa provides an absorption surface of approximately 150 cm^2^, is richly vascularized, and exhibits reduced enzymatic metabolism compared to the gastrointestinal system [16]. Moreover, it allows direct access to the central nervous system (CNS) via the olfactory and trigeminal pathways, bypassing the blood–brain barrier (BBB), a major limiting factor for many drugs targeting the neurological system [17]. In this context, nanostructured systems such as nanoemulsions and microemulsions have been explored for their ability to prolong residence time in the nasal cavity, enhance drug permeation, and protect the active compound from local degradation. The incorporation of mucoadhesive polymers, such as chitosan or hyaluronic acid, can further intensify these effects by increasing the contact time with the mucosa, thereby improving absorption and therapeutic efficacy [15,18,19].

In line with advances in pharmaceutical innovation, scientometrics has emerged as an essential tool for examining global scientific output. This field focuses on the quantitative analysis of scientific activity through indicators such as the volume of published articles, impact factor, h-index, authorship collaborations, and citation metrics [20]. By utilizing various databases such as Scopus and Web of Science, along with modern computational tools, scientometrics enables the identification of research trends, the discovery of emerging fields, the analysis of collaboration networks, and the support of government policies for research promotion [21,22]. The application of this methodology to the field of nanostructured systems, such as intranasal nanoemulsions, enables not only the observation of the scientific expansion of the field but also the identification of opportunities for innovation and multidisciplinary collaboration [23,24].

Thus, understanding the scientific evolution of nano- and microemulsions for intranasal use through scientometric methods serves as a valuable tool for researchers to highlight the relevance of these systems within current pharmaceutical nanotechnology and to identify promising areas in which to focus their efforts [25]. While bibliometric analysis does not directly assess the clinical efficacy or pharmacological performance of drug delivery systems, it enables the identification of publication patterns, to identify emerging trends, knowledge gaps, and areas for improvement, thereby guiding future investigations aimed at developing safer, more effective, and more accessible intranasal drug delivery systems [25,26]. Given the growing interest in non-invasive and targeted delivery approaches, particularly for treating neurological disorders and employing controlled release technologies [18,27,28], this analysis can provide valuable support for scientific decision-making and the promotion of research funding.

## 2. Materials and Methods

### 2.1. Materials

Bibliometric analyses were carried out using the Bibliometrix package (version 4.1.3, developed by Massimo Aria and Corrado Cuccurullo, Naples, Campania, Italy), implemented in the statistical environment R (version 4.4.3, R Core Team, Vienna, Austria). For network visualization and mapping, VOSviewer (version 1.6.20, developed by Nees Jan van Eck and Ludo Waltman, Leiden, South Holland, The Netherlands) was employed. For complementary statistical and graphical treatment, the software Origin 6.0 (OriginLab Corporation, Northampton, MA, USA) was used.

### 2.2. Data Collection and Screening

Data collection was conducted in May 2025 using the Scopus database (https://www.scopus.com, accessed on 17 May 2025), which is internationally recognized for its comprehensive coverage and relevance in indexing high-impact scientific journals. This platform includes the CiteScore, SCImago, Journal Rank (SJR), Source-Normalized Impact per paper (SNIP), the Field-weighted Citation Impact and PlumX Metrics, ensuring multidisciplinary coverage of publications. The choice of Scopus as the primary data source is justified not only by its broad acceptance and credibility in scientometric research, but also by its compatibility with bibliometric analysis tools. Unlike other databases, Scopus allows for the export of metadata in formats such as .bib and .csv, which are essential for use with platforms like Bibliometrix (R/Biblioshiny) and VOSviewer. Moreover, attempts to integrate data from multiple databases, such as Web of Science and PubMed, proved unfeasible due to incompatibility issues, missing reference fields, and metadata loss during file merging, which compromised the reliability of the results. These technical limitations have also been reported in previous bibliometric studies, many of which opt to use a single database to ensure the accuracy and consistency of the analysis [25].

Table 1 outlines the strategy used for identifying and selecting the studies included in this analysis. The search criteria were as follows: (1) the use of the terms “Nanoemulsion OR Microemulsion” combined with “Nose-to-brain OR Intranasal” in the topic fields; (2) a publication date range from 1 January 2004, to 31 December 2024; (3) inclusion of original research articles; (4) exclusion of reviews, book chapters, retractions, letters to the editor, conference proceedings, errata, editorials, short surveys, and abstracts; and (5) removal of duplicate records if necessary. The initial search retrieved 614 publications prior to applying the time filter. After limiting the results to the defined time frame, 582 records remained. Of these, 379 were original research articles included in the final analysis. Although this study did not explicitly exclude articles unrelated to pharmaceutical nanotechnology, such as those focused on food science, environmental studies, or forensic applications, the specificity of the keywords “intranasal” and “nose-to-brain” effectively prevented the inclusion of such content. Furthermore, all retrieved records were carefully reviewed, and no articles related to food, environmental, or forensic contexts were identified, as can be verified in the Supplementary Material “Excel intranasal Scopus.” Duplicate records were identified and, if necessary, removed using the R statistical environment (version 4.3.3, manufacture, city, if any state, country) through custom programming scripts. The complete list of included publications is provided in the Supplementary Material.

### 2.3. Bibliometric Analysis

**Figure 1:**
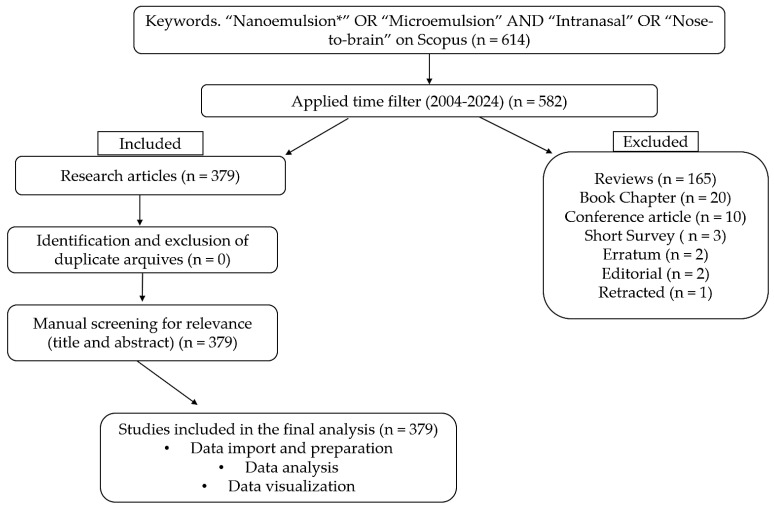
PRISMA-style flow diagram illustrating the study selection process.

All data obtained were processed in the R statistical environment (version 4.3.3) using the Bibliometrix package, which is developed in R and widely employed in bibliometric studies. This package provides the Biblioshiny graphical interface, which enables interactive navigation and facilitates information filtering, as well as the definition of indicators, methods, and analytical parameters. This study was conducted in three main stages: (1) data import and preparation from Scopus (Supplementary Material “Excel intranasal Scopus”), during which bibliographic records were loaded and converted into a data frame in R (Supplementary Materials R Script to biblioshine); (2) data analysis, which was divided into three phases: (i) descriptive analysis of the bibliographic dataset; (ii) construction of bibliometric networks, including bibliographic coupling, co-citation, co-authorship, and keyword co-occurrence (Supplementary Material Biblioshiny_Report); and (iii) application of normalization techniques. These analyses were performed using tools available within the Biblioshiny interface, which automatically generates these outputs based on Scopus data reports.; and (3) data visualization, with mapping of the conceptual structure and relationship networks through graphical representations generated from the previous analyses. Figure 1 presents a PRISMA-style flow diagram that illustrates the criteria for inclusion and exclusion, as well as the data sources and the methodological approach used for data analysis.

During the preparation of this work, the author(s) used ChatGPT (GPT-5, OpenAI, 2025) to enhance the readability and language of the manuscript. After using this tool/service, the author(s) reviewed and edited the content as needed and take full responsibility for the content of the published article.

## 3. Results and Discussion

### 3.1. Volume and Publication Trends

**Figure 2:**
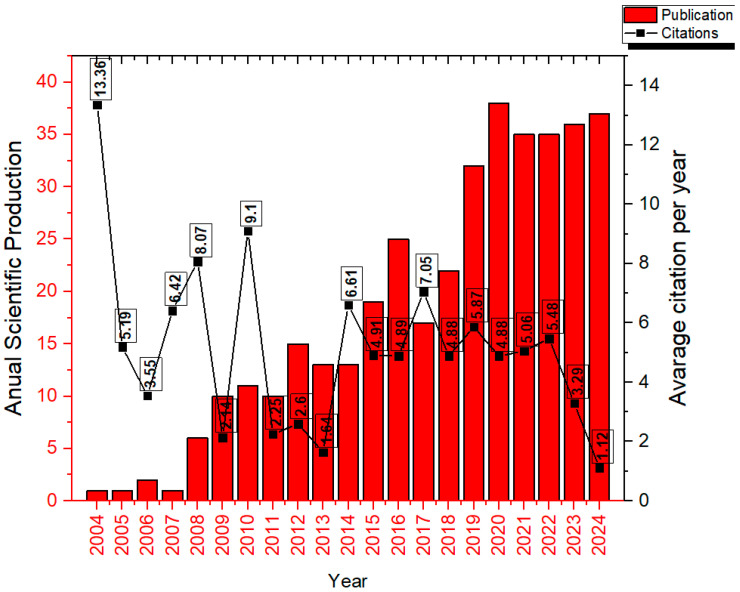
Annual scientific output of publications on intranasal nano- and microemulsions and average citations per article from 2004 to 2024.

A total of 379 manuscripts involving intranasal nano- and microemulsions were identified; of these, 377 were published in English and 2 in Arabic. Figure 2 displays the number of publications per year alongside the average number of citations.

Figure 2 presents the annual scientific output (red bars) and the average number of citations per year (black line) from 2004 to 2024. The annual growth rate of publications was approximately 20.55%. A progressive increase in publication volume is observed starting in 2009, with notable peaks in 2018 and a subsequent stabilization at approximately 30 publications per year. The year 2020 recorded the highest number of publications (37), although it had a lower average citation rate (4.88). Conversely, years with fewer publications, such as 2004 and 2010, exhibited higher average citation counts (13.36 and 9.1, respectively). These findings suggest a continuous growth in publication volume, accompanied by a decline in average article impact in more recent years.

The annual average number of citations was 5.06, while the median was 4.91. It was observed that after 2019, the number of publications increased compared to the first ten years analyzed; however, the number of citations decreased. This effect is explained by Frachtenberg, who noted that the median number of citations per article tends to increase almost linearly, with only a few publications reaching peak citation rates within five years and even fewer exhibiting exponential growth. In general, articles begin to be cited between 9 and 12 months after publication, and the average time required to reach a given number of citations tends to increase in near-proportional fashion [29].

The same author also evaluated the average citation timeline and found that the mean time for an article to receive its first citation was 6.24 months, longer than the time required to reach the next following citation, which was 2.91 months. This interval continues to decrease gradually, with an average of only 1.86 months needed to reach the tenth citation [29]. This pattern is understandable, as articles begin to circulate more widely within the scientific community, becoming increasingly recognized and cited by a growing number of researchers. Consequently, it is expected that more recent publications, such as those from 2023 and 2024, still show a lower number of citations, as there has not yet been sufficient time for these works to gain broad visibility and be substantially incorporated into ongoing research.

The production and use of nano- and microemulsions for intranasal delivery increased by over 1150% in just ten years, when comparing 2006 to 2016. This surge can be attributed to intensified research into the intranasal route for delivering drugs that act on the CNS, particularly for the treatment of brain tumors, Parkinson’s disease, Alzheimer’s disease, depression, among others [2,30,31]. The investigations involve delivery mechanisms, drug deposition sites, and uptake pathways.

The intranasal route is particularly attractive for nose-to-brain drug delivery, as it enables the administration of therapeutics directly to the CNS, bypassing the BBB via the olfactory or trigeminal nerves. Moreover, since it does not involve direct access to the systemic circulation, this route is associated with a reduced incidence of adverse effects [28,32].

The BBB plays a crucial role in protecting the brain, acting as a defense mechanism against potentially harmful substances and microorganisms. It is composed of extremely tight intercellular junctions, along with additional protective mechanisms such as efflux pumps and glycoproteins [33,34]. Although these mechanisms are essential for neural protection, they hinder the effective delivery of drugs to the brain. In this context, intranasal administration emerges as a promising strategy, as it enables the partial bypassing of these barriers, thereby facilitating targeted therapeutic delivery to the CNS [18].

The distribution pathways of nanoparticles or other drugs following intranasal administration occur primarily via the olfactory and trigeminal routes, which are the main conduits for CNS delivery through this route. Compounds cross the nasal epithelium to reach the lamina propria, predominantly through extracellular transport, and subsequently reach the CNS mainly via bulk flow through perineural and perivascular spaces [35].

The nasal cavity is composed of three main regions: vestibular, olfactory, and respiratory. Nasal hairs and the mucosal lining constitute an effective first line of defense for the respiratory tract. The vestibular region, located just inside the nostrils, is sparsely vascularized and contains non-ciliated epithelial cells along with nasal hairs, resulting in minimal drug absorption due to its small surface area, approximately 0.6 cm^2^ [35,36].

The respiratory region, which comprises the largest portion of the nasal cavity, approximately 130 cm^2^, is highly vascularized, making it an efficient surface for drug absorption. This region consists of roughly 20% mucus-producing goblet cells and 80% ciliated epithelial cells, which are interconnected by tight junctions. Together, these cells perform mucociliary clearance (MCC), a defense mechanism that traps and transports particles through the mucus layer. The MCC transit time is approximately 20 min, making it a critical factor to consider in intranasal drug administration. The trigeminal nerve is also present in this region, contributing to drug transport toward the CNS [35,37].

The olfactory region is located in the upper part of the nasal cavity, beneath the cribriform plate, which contains small perforations that facilitate access to the CNS. This region accounts for approximately 10% of the total nasal surface area (~15 cm^2^) and is highly vascularized. The olfactory epithelium is innervated by both the olfactory and trigeminal nerves. The transfer of compounds from the nose to the brain through the olfactory region may occur via multiple mechanisms, as discussed later in this work [28,35,36,37].

For nose-to-brain drug delivery to be effective, it is essential that the drug remains in the nasal cavity for a sufficient period to allow for absorption [18]. The term microemulsion refers to homogeneous, transparent, and thermodynamically stable systems composed of oil, water, and surfactants [38]. Microemulsions possess several distinctive characteristics that support drug delivery through this route, such as viscosity, thermodynamic stability in response to temperature, and the choice of oil phase, which can enhance drug absorption, all of which are critical [5,8,39,40]. Most importantly, particle size plays a key role; microemulsions with droplet sizes between 50 and 100 nm are particularly advantageous for drug delivery, as they facilitate tissue perfusion, enable cellular compartment access, and promote targeted drug release at the desired site [5,7,18,41].

The optical transparency of these systems suggests that their internal structures have dimensions smaller than 100 nanometers, as confirmed by various analytical techniques, including small-angle X-ray scattering (SAXS), small-angle neutron scattering (SANS), diffusion-based nuclear magnetic resonance (NMR), and cryogenic electron microscopy (cryoTEM and cryoSEM) [5]. Additionally, the term nanoemulsion was later introduced to describe systems with droplet sizes ranging from 10 to 1000 nm, but with different characteristics in terms of preparation, thermodynamics, and stability [30]. These small dimensions provide a large interfacial area, which is spontaneously achieved due to the extremely low interfacial tension. Under such low-tension conditions, the typical need for spherical structures, usually adopted to minimize the surface area to volume ratio, becomes less dominant, allowing oil and water domains to assume geometries other than conventional droplets [5,40]. For this reason, the term microemulsion may be considered a misnomer, as these structures are actually within the nanometric range and often do not involve true droplets. Nevertheless, the term has persisted and is commonly used to describe the systems with the aforementioned characteristics [5,39].

Depending on the proportions of water, oil, and surfactant used, microemulsions can be classified as either oil-in-water (O/W) or water-in-oil (W/O) systems. In O/W microemulsions, the oil phase is dispersed within the aqueous phase and stabilized by the action of surfactants [41,42]. It is important to note that the formation of microemulsions occurs only within a specific and well-balanced compositional range, often requiring the combination of multiple surfactants. This formation is influenced by several factors, including the nature of the oil phase, salt concentration in the aqueous phase, and system temperature. Moreover, the addition of co-solvents, such as medium-chain alcohols, is frequently necessary, making the development of a microemulsion a technically challenging process [5,6].

The marked increase in research output on intranasal nano- and microemulsions over the past two decades reflects a convergence of technological advances, unmet clinical needs, and evolving therapeutic strategies for CNS disorders. Growing recognition of the limitations imposed by the blood–brain barrier on conventional drug delivery has driven interest in alternative administration routes capable of achieving direct nose-to-brain transport. Intranasal nano- and microemulsions, with their capacity to enhance solubility, stability, and bioavailability in poorly water-soluble compounds, offer a compelling solution, particularly for neurologically active agents with restricted BBB permeability. The surge in publications from 2009 onward aligns with broader trends in nanomedicine, including the refinement of lipid-based carriers, improved characterization techniques, and a shift toward patient-friendly, non-invasive delivery systems. Moreover, the rising global burden of neurodegenerative and neuropsychiatric diseases, coupled with the demand for rapid-onset therapeutics in acute neurological conditions, has further amplified the relevance of this approach. This sustained research interest is therefore underpinned by both translational potential and the strategic role of intranasal nano- and microemulsions in addressing pharmacokinetic and delivery challenges that remain unsolved by conventional formulations.

### 3.2. Scientific Production by Country

**Figure 3:**
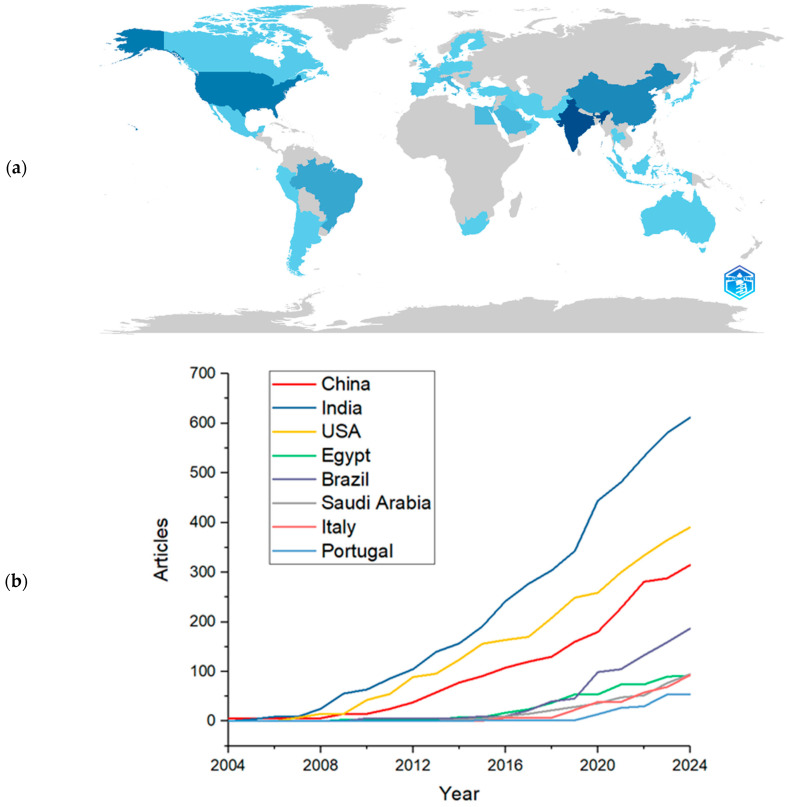
(**a**) Analysis of global scientific production over two decades, highlighting the 20 countries with the highest number of publications in the period. (**b**) Growth in the number of articles from the 10 most prolific countries in this field. Source: Bibliometrix [26,43] through Scopus data, 2025.

A total of 44 countries were identified in this survey. Statistical analyses were conducted based on the frequency of author appearances, considering the countries or regions associated with their affiliated institutions. Figure 3a presents the twenty countries with the highest number of publications in this field, accounting for all contributing authors.

The Bibliometrix software calculated the publication frequency, indicating how much each country published about the theme during the analyzed years. The graphical interface of the Bibliometrix package, publication frequency is automatically calculated by counting the number of occurrences of each entity (such as author, country, institution, journal, or keyword) within the imported bibliographic dataset. Once the parameter of interest is selected, the software tallies how often each element appears over the analyzed period, with optional temporal filters and normalization. The results are displayed in tables and graphical formats, highlighting publication trends and enabling the identification of the most productive authors, countries with the highest publication output, the most frequent journals, and other key bibliometric indicators [26,43]. As shown in Figure 3a, the intensity of the blue color corresponds to the frequency of publications in each country, darker shades indicate a higher number of occurrences of publication. The specific number of articles per country will be detailed in following sections. India leads in publication volume, with 612 articles, in frequency, which is the number of occurrences of each entity (such as author, country, institution, journal, or keyword) within the imported bibliographic dataset, followed by the United States with 391, China with 315, Brazil with 187, Saudi Arabia with 95, Italy with 93, Egypt with 92, and Portugal with 54. The remaining countries each contributed fewer than 50 articles. Figure 3b further highlights the growth in scientific output across these countries.

A notable difference is observed between the first and second positions: India published approximately 56% more articles than the United States. This phenomenon can be attributed to several factors. Firstly, India has a very large population and a rapidly expanding academic base, with a significant increase in the number of universities, research centers, and graduate programs. This structural growth naturally drives the country’s scientific output. In addition, Indian academic policies often link career advancement and funding opportunities to the number of published articles, which strongly incentivizes productivity, frequently resulting in greater emphasis on quantity rather than quality [44].

India’s high population density may also be a relevant factor, as a greater number of individuals may have access to academic institutions within a relatively smaller geographic area, especially when compared to the territorial extension of the United States. Additionally, the quality of the selected publications should not be disregarded. All included articles were indexed in reputable databases comprising journals with moderate to high impact factors, ranging higher than 3. It is important to note that peer-reviewed acceptance indicates scientific merit, regardless of whether the journals charge publication fees.

The trajectory of scientific production growth across different countries over the past 20 years, as shown in Figure 3b, highlights India as the country with the highest number of publications in the period, demonstrating sharp and continuous growth since 2008, reaching over 600 articles by 2024. The United States ranks second, maintaining a stable and significant output since the early years analyzed, with a notable increase after 2010 and surpassing 400 publications by 2024. China, in turn, exhibits a more linear but steady growth trend, reaching approximately 300 publications within the same timeframe.

Brazil also shows a relevant performance, with its scientific production in this field becoming more prominent from 2012 onward and continuing to grow steadily, surpassing 200 publications by 2024. As an emerging country, Brazil, similar to India, which leads the ranking in publication volume, has shown a steady increase in the number of scientific publications over recent years. This growth can be attributed to the expansion of research activities within public universities, a rise in the number of researchers supported by scholarships, and a growing interest in technological fields. Additionally, support for research funding and publication incentives has played a key role, especially considering that many universities face limitations due to the lack of dedicated publication funds. CAPES (Coordination for the Improvement of Higher Education Personnel), a major Brazilian federal agency, has reinforced these efforts by promoting initiatives that enable researchers to publish high-quality, impactful studies.

Egypt, Saudi Arabia, Italy, and Portugal exhibit later and more gradual increases, with a notable rise in publication activity beginning around 2016–2018. Although these countries have lower overall output, they demonstrate increasing interest and engagement with the topic.

It is important to emphasize that investment in technology is essential in the pharmaceutical field, particularly given that universities serve as primary centers for scientific research worldwide. In emerging countries, where private sector investment is often limited, universities are largely responsible for sustaining the research infrastructure necessary for technological advancement. Many of these institutions have established their own patent protections, which not only support researchers in the development and protection of innovations but also strengthen the university’s role as a generator of practical and commercial solutions. Unfortunately, much of the research funding in these contexts relies heavily on government support, which has been subject to significant budget cuts in recent years. Nevertheless, researchers continue to make remarkable progress despite limited resources, often achieving exceptional results under constrained conditions.

### 3.3. Afiliations

**Figure 4:**
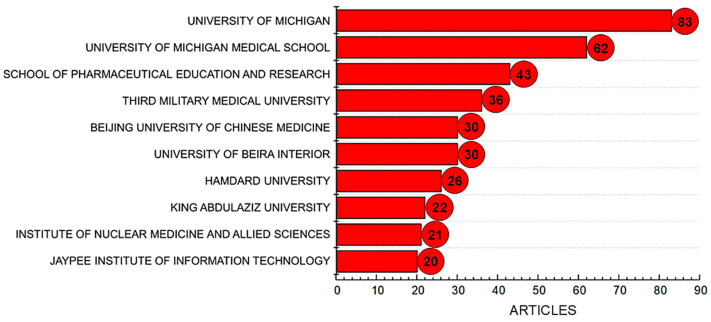
Most relevant institutions in global scientific production on intranasal nanoemulsions from 2004 to 2024. Source: Bibliometrix through Scopus data, 2025.

Figure 4 illustrates the ten most relevant institutions in terms of scientific output related to the topic analyzed, comprising contributions from a total of 441 academic institutions. Among these, the University of Michigan stands out as the leading institution, with 83 publications, followed by the University of Michigan Medical School, which accounts for 62 articles.

The University of Michigan stands out as the leading institution in this field, likely due to its long-standing tradition in pharmaceutical sciences and biomedical research. Its strong infrastructure, multidisciplinary research environment, and substantial funding, particularly from national agencies such as the NIH, enable the development of advanced drug delivery systems, including intranasal nano- and microemulsions. Moreover, the presence of dedicated research centers focused on nanomedicine, pharmacokinetics, and translational medicine fosters continuous innovation and high publication output.

The School of Pharmaceutical Education and Research in India ranks third, with 43 publications. Other notable institutions include the Third Military Medical University in China (36 articles), Beijing University of Chinese Medicine and the University of Beira Interior in Portugal (both with 30 articles), and Hamdard University in Pakistan (26 articles). King Abdulaziz University in Saudi Arabia (22 articles), the Institute of Nuclear Medicine and Allied Sciences in India (21 articles), and the Jaypee Institute of Information Technology in India (20 articles) also rank among the most productive.

The data presented reflect not only the growing global interest in intranasal nano- and microemulsion systems, but also the strategic positioning of emerging and established institutions in advancing this field. The diversity in geographic distribution underscores the increasing accessibility of research tools and international collaboration, while the concentration of publications in specific institutions suggests the presence of specialized research groups with dedicated funding and infrastructure. Moreover, the prominence of institutions from countries like India, China, and Pakistan highlights the essential role of developing nations in shaping the future of pharmaceutical innovation. This global engagement is both inspiring and encouraging, it reveals a shared commitment to scientific progress despite regional disparities in resources. It also reflects the determination of researchers worldwide to overcome limitations and contribute meaningfully to health-related advancements. Ultimately, these findings emphasize that impactful science knows no borders, and that innovation can flourish wherever there is dedication, collaboration, and institutional support.

### 3.4. Scientific Journals

A total of 165 scientific journals published studies related to the use of nano- and microemulsions for intranasal delivery. Table 2 presents the most frequently recurring journals in disseminating advances in this area, organized in descending order according to the number of publications. The table also includes the total number of citations (TC) and the H-index for each journal. These data are particularly relevant for researchers seeking to identify the most suitable journals for submitting their work.

The scientific journals that stand out the most in terms of publication volume in this field are the *International Journal of Pharmaceutics*, with 27 articles, and the *Journal of Drug Delivery Science and Technology*, with 18. The former stands out significantly in terms of impact, with a total of 1783 citations and an H-index of 18, making it one of the leading references for researchers in the area. The latter has accumulated 513 citations and an H-index of 14, also indicating broad relevance and visibility.

Three other journals, *AAPS PharmSciTech*, *Drug Delivery*, and *Pharmaceutics*, each published 14 articles, although their impact varied. *Drug Delivery* achieved the highest number of citations among them (946), followed by *AAPS PharmSciTech* (492) and *Pharmaceutics* (442), with H-indices of 14, 10, and 9, respectively. These data highlight not only the editorial productivity but also the scientific influence of these journals.

*Molecular Pharmaceutics* published nine articles and received 245 citations, with an H-index of 8, demonstrating a significant contribution despite a lower number of publications. The journal *Vaccine* published 8 articles, totaling 216 citations and an H-index of 7, maintaining a strong proportional performance.

Among the journals with fewer publications but notable impact are *Artificial Cells*, *Nanomedicine and Biotechnology*, and *Current Drug Delivery*, both with seven articles. The former accumulated 272 citations and has an H-index of 7, while the latter reached 204 citations and an H-index of 6. Lastly, *Drug Development and Industrial Pharmacy* published 6 articles, received 248 citations, and also holds an H-index of 6. These findings underscore the diversity of relevant scientific journals in the field and serve as a useful guide for publication and literature consultation.

The prominence of journals such as the *International Journal of Pharmaceutics*, *Journal of Drug Delivery Science and Technology*, and *Drug Delivery* in publishing studies on intranasal nano- and microemulsions can be attributed to their strong focus on drug delivery systems, formulation science, and translational research. These journals provide a target that bridges pharmaceutical innovation and clinical applicability, making them an outlet for studies in this field. Furthermore, their relatively high impact metrics suggest a readership engaged in applied pharmaceutical research, fostering citation and visibility. The presence of specialized journals such as *Pharmaceutics* and *AAPS PharmSciTech* further reflects the maturation of the field and the diversification of publication venues for nanotechnology-based delivery approaches. Given this landscape, it becomes clear that journal selection in this niche is shaped not only by scope and impact factor but also by alignment with interdisciplinary trends in drug development. As intranasal delivery continues to gain traction for targeting the CNS and improving patient compliance, one might ask if the current publication trends are keeping pace with the evolving complexity and innovation in this domain.

### 3.5. Most-Cited Manuscripts

The relevance of a scientific article is often assessed by the number of times it is cited by other studies. In this context, the five most influential publications on nano- and microemulsions for intranasal administration were identified, as presented in Table 3. These works primarily focus on the development of nano- and microemulsion-based systems aimed at effective nasal drug delivery, with an emphasis on improving absorption, bioavailability, and therapeutic efficacy. Additionally, they highlight innovations in rapid-release formulations, simplified production methods, and the use of accessible technologies for the characterization and evaluation of nano- and microemulsions.

The most-cited article is titled “Intranasal delivery to the CNS: Mechanisms and experimental considerations”, published in the *Journal of Pharmaceutical Sciences* in 2010, with an impressive total of 1043 citations. This journal publishes research focused on physical and biological barriers to drug action, as well as strategies to optimize efficacy, safety, accessibility, and cost. It covers fields such as pharmaceutical technology, pharmacokinetics, nanoscience, biochemistry, medicinal chemistry, and innovative drug delivery systems. The article explores intranasal administration as a promising strategy for delivering drugs to the CNS, overcoming the limitations imposed by the BBB. It describes this route as non-invasive and capable of enabling rapid delivery of compounds to the CNS while minimizing systemic exposure and adverse effects. The main anatomical pathways involved, such as the olfactory and trigeminal nerves, vasculature, cerebrospinal fluid, and lymphatic system, are discussed in detail as contributors to the transport of drugs from the nasal cavity to the brain. Additionally, the study highlights that formulation characteristics (such as pH, osmolarity, presence of mucoadhesive agents or permeation enhancers), the administered volume, head position, and application technique all directly influence the effectiveness of intranasal delivery. The review emphasizes the importance of carefully considering these parameters in the development of intranasal therapies and calls for further research to optimize this therapeutic approach [45].

The second-most-cited article, with 421 citations, is titled “Intranasal nanoemulsion based brain targeting drug delivery system of risperidone”, published in the *International Journal of Pharmaceutics* in 2008. The study aimed to develop and evaluate risperidone-loaded nanoemulsions for intranasal administration to optimize brain delivery. Two formulations were compared: a simple nanoemulsion (RNE) and a mucoadhesive nanoemulsion (RMNE). Both were characterized in terms of drug content–98.86% for RNE and 99.12% for RMNE–pH (ranging from 3.5 to 6.5), particle size (15.5–16.7 nm), and zeta potential. Using technetium-99m-labeled risperidone, the study assessed brain and blood distribution in rats following both intranasal and intravenous administration. The intranasal RMNE formulation demonstrated greater brain uptake and higher direct transport efficiency (DTE%) and direct transport percentage (DTP%) compared to the other formulations. These results indicate its effectiveness in crossing the blood–brain barrier and enhancing risperidone delivery to the CNS [46].

The third-most-cited article is titled “Crossing the Blood–Brain Barrier: Recent Advances in Drug Delivery to the Brain”, published in 2017 in *CNS Drugs* by Springer Nature, with 345 citations. This article discusses the challenges and advances in treating CNS disorders, highlighting the BBB as the main obstacle to drug entry into the brain. Although many promising therapeutic candidates have been identified, their clinical efficacy is often limited by the difficulty in crossing this protective barrier. To address this limitation, researchers have focused on reformulating existing drugs and developing targeted delivery strategies. The article explores physiological approaches such as receptor-mediated transport and efflux inhibition, as well as the use of nanostructured systems including liposomes, nanoparticles, nanoemulsions, and dendrimers. It also examines alternative administration routes, such as intranasal delivery, and techniques aimed at transiently opening the BBB. The review compiles recent developments, particularly from 2015 and 2016, providing a comprehensive overview of the most promising strategies for enhancing drug access to the CNS [47].

The fourth-most-cited article, with 342 citations, was published in *Drug Delivery and Translational Research* in 2022 and is titled “Intranasal drug delivery: opportunities and toxicologic challenges during drug development.” This study highlights the growing interest over the past decade in using the intranasal route for drug administration, particularly in the treatment of neurological disorders. The nasal route offers several advantages over traditional systemic approaches, such as non-invasive application, rapid onset of action, and a lower incidence of adverse effects due to more targeted delivery. The article discusses the anatomical, histological, and physiological foundations of this route, in addition to listing the drugs already approved for intranasal use. It also addresses formulation challenges and concerns regarding local toxicity, emphasizing the need for optimization in these areas to ensure safety and efficacy in the development of new intranasal therapeutics [28].

The fifth-most-cited article is titled “Preparation of nimodipine-loaded microemulsion for intranasal delivery and evaluation on the targeting efficiency to the brain”, with 294 citations, published in 2004 in the *International Journal of Pharmaceutics*. The study aimed to develop an oil-in-water microemulsion for the intranasal delivery of nimodipine (NM), with the goal of enhancing its solubility and promoting greater brain uptake. Various systems with non-ionic surfactants and different oils were tested, with the optimal formulation consisting of Labrafil M 1944CS, Cremophor RH 40/ethanol, and water. This microemulsion demonstrated good drug solubility (6.4 mg/mL), small droplet size (~30 nm), and no evidence of nasal toxicity. In rat models, the intranasally administered formulation showed a bioavailability of 32% and a threefold increase in uptake in the olfactory bulb compared to intravenous administration. Drug distribution in the brain and cerebrospinal fluid was also significantly higher with nasal delivery, indicating that the microemulsion represents a promising strategy for the use of NM in therapies targeting neurodegenerative diseases [48].

The high citation rates of these articles can be attributed to three main factors. First, their early publication dates, particularly those from 2004, 2008, and 2010, allowed them to accumulate citations over a longer period, establishing them as foundational references in the field. Second, they were published in high-impact journals with strong visibility in pharmaceutical sciences, such as the *Journal of Pharmaceutical Sciences* and the *International Journal of Pharmaceutics*, which significantly increases their exposure to a global research audience. Third, these studies addressed critical knowledge gaps by offering experimental evidence on mechanisms of intranasal transport, comparative pharmacokinetics, and formulation strategies, all of which remain central to drug delivery research today.

Dhuria et al. 2010 [45] provide a mechanistic and experimental framework for understanding nose-to-brain transport pathways, emphasizing critical variables such as dosing parameters, formulation attributes, and administration techniques. Although not specific to nano- or microemulsions, this work has become foundational by offering a conceptual basis that underpins subsequent formulation design. Kumar et al. 2008 [46] represent a methodological milestone by demonstrating a nanoemulsion-based system for risperidone, integrating high-energy emulsification, which is already used in laboratory practice, physicochemical optimization, and in vivo brain-targeting evaluation, offering a complete translational pipeline from formulation to pharmacodynamic assessment. Patel et al. 2017 [47] broaden the scope with a comprehensive synthesis of strategies to overcome the blood–brain barrier, situating intranasal nanoemulsions within a wider technological landscape and influencing how future research selects and combines delivery platforms. Keller (2022) [28] contributes by critically analyzing toxicological considerations during intranasal drug development, bringing attention to safety profiling and regulatory compliance, an area often underrepresented in formulation-focused studies, yet essential for clinical translation. Finally, Zhang et al. 2004 [48] deliver a detailed formulation and evaluation of nimodipine-loaded microemulsions, including systematic droplet size control, stability assessment, and quantification of brain-targeting efficiency, providing a methodological template that blends physicochemical rigor with biological validation.

Beyond the analyses, these publications resonate with researchers for offering clarity, practicality, and innovation at a time when intranasal administration is emerging as a serious alternative for CNS therapies. There is also a sense of continuity, as these works laid the foundation for a decade of translational research and inspired subsequent studies across various disciplines. Furthermore, the products developed in the research, often pioneering or introducing innovative technologies, may be targets for investors willing to patent the product and begin commercial trials.

Nevertheless, these articles gained influence not simply by being early or well placed, but by asking and answering the right questions at the right time. The critical reflection then is, as the field becomes more crowded and technologically advanced, are current studies still driven by fundamental questions with broad translational value, or are we losing sight of what truly builds lasting impact. The Bibliometrix study helps to show this impact due to reuniting the finest and the most viewed publications.

### 3.6. The Keyword Co-Occurrence Network

The analysis of keywords allows for a comprehensive understanding of the scope, subfields, and main themes within a scientific domain. Authors use keywords to highlight the core concepts of their studies, facilitating the retrieval of the most relevant publications in investigations involving digital imaging and smartphone use. In this study, a total of 1009 author-assigned keywords were identified, with 24 of them occurring 10 times or more.

**Figure 5:**
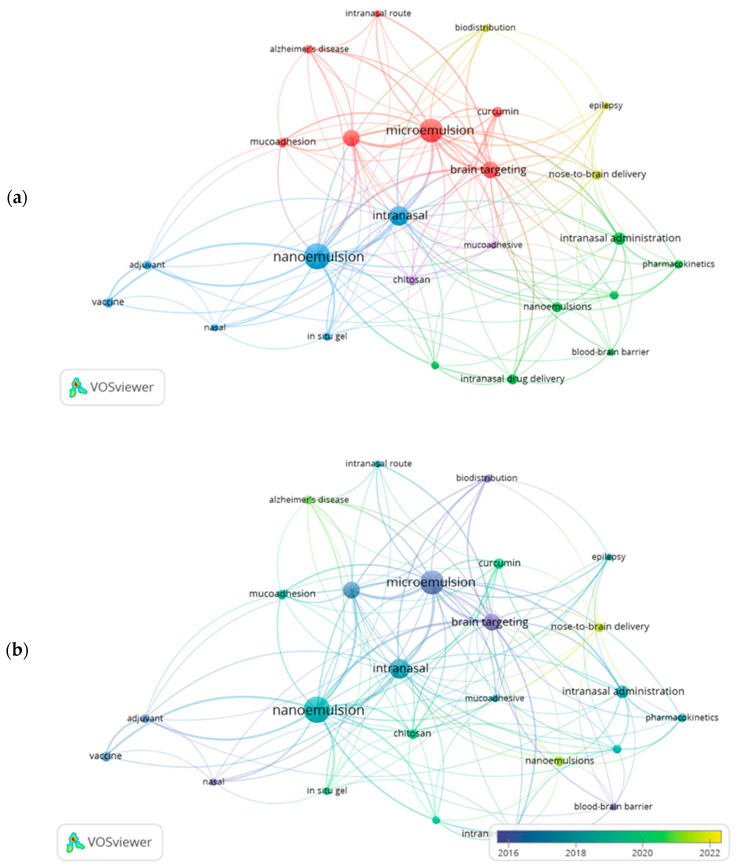
(**a**) Co-occurrence network of the top 20 keywords. (**b**) Keyword co-occurrence network by time. Source: The author, through VOSviewer, 2025.

Figure 5a presents the network of the 20 most frequently used keywords, followed by Figure 5b, which illustrates their co-occurrence over time.

The size of each circle reflects the frequency with which a given keyword was used in the analyzed publications—larger circles represent more frequently used terms. The links between the circles indicate co-occurrence, meaning that the keywords appeared together in one or more articles. The thickness of these links is proportional to the frequency of their co-occurrence. Keywords that are positioned closer to one another exhibit a stronger relationship and tend to appear together in publications, forming thematic clusters.

To gain a more detailed understanding of the significance of each term within the keyword network, three centrality metrics are applied. Betweenness measures how much a node acts as a bridge between different regions of the network; closeness assesses how close a term is to all other terms; and PageRank estimates the relevance of a node based on the quality of its connections. The data related to these metrics are presented in Table 4.

The co-occurrence analysis of keywords enables the identification of major thematic focuses and semantic relationships in the field of nano- and microemulsions applied to intranasal drug delivery. The term “nanoemulsion” stands out as one of the most central nodes in the network, presenting the highest betweenness (402.621) and PageRank (0.128) values, highlighting its prominent role as a cross-cutting technology of interest in various therapeutic contexts. The high centrality of this term suggests that nanoemulsions serve as a key point of articulation across diverse topics, from pharmaceutical formulations to therapeutic targets, being widely recognized for their versatility in encapsulating and delivering active compounds.

Included in Cluster 4, the term “nanoemulsion” shows strong connections with modern therapeutic strategies, especially in the context of targeted delivery systems, primarily at the interface with the intranasal route. The term “microemulsion”, also with high centrality values (betweenness of 376.261 and PageRank of 0.128), is part of Cluster 1, suggesting a thematic link to other fundamental concepts of intranasal pharmaceutics, such as “intranasal”, “intranasal delivery”, and “drug delivery”.

The prominence of the terms “nanoemulsion” and “microemulsion” in the co-occurrence network can be explained by three converging factors. First, both terms represent new fronts of technologies in drug delivery, and their continued presence across clusters reflects their versatility in pharmaceutical innovation, being compatible with many kinds of drugs. Second, their association with the intranasal route, an increasingly investigated alternative for bypassing the blood–brain barrier, positions these keywords at the core of current strategies for CNS targeting. Third, the high centrality values of “microemulsion” in Cluster 1 and “nanoemulsion” in Cluster 4 demonstrate their bridging role between formulation science and therapeutic application, linking fundamental and translational research.

These findings resonate with the scientific community’s growing interest in precise, non-invasive delivery systems and reflect an ongoing effort to translate complex formulations into clinically viable treatments. There is also a sense of intellectual convergence where different research paths meet at shared technological platforms. The critical question moving forward is whether the field will continue to innovate on these established systems or shift toward entirely new paradigms.

To further clarify, from the 379 articles retrieved in Scopus, 227 referred to “nanoemulsions” and 152 to “microemulsions” (see Supplementary archive of Scopus data). As illustrated in Figure 5b, “microemulsion” appears predominantly around 2016, while “nanoemulsion” and “nanoemulsions” cluster more strongly between 2018 and 2022. This distribution confirms that both terms contribute meaningfully to the field, reflecting the growing scientific interest in these technologies, even when their definitions are sometimes used interchangeably.

Cluster 1 generally groups the main terms related to the administration route, with “intranasal” (betweenness 130.565; PageRank 0.078) serving as a central node in the thematic area of nasal drug delivery. Terms like “intranasal delivery” and “intranasal drug delivery” also appear within this cluster with significant PageRank values (0.047 and 0.021, respectively), underscoring the growing focus on the nasal route as a promising alternative for delivering bioactive compounds to the CNS by bypassing the blood–brain barrier. The inclusion of “nasal” and “mucoadhesion” in this cluster further highlights the importance of physicochemical properties, such as mucoadhesiveness, for formulation success.

Still within the brain-targeting perspective, the term “brain targeting”, included in Cluster 1, shows a high PageRank value (0.087), indicating its relevance in the theme of intracerebral targeting, frequently associated with colloidal systems such as nanoemulsions. The appearance of the term “brain delivery” in Cluster 2, although with a lower centrality score (PageRank 0.016), complements the notion that the intranasal route is increasingly explored as a non-invasive pathway to efficiently reach the brain.

Among the 379 analyzed articles, 129 targeted the brain, addressing treatments for conditions such as cancers [49,50], Alzheimer’s disease [51,52,53], Parkinson’s disease [54,55,56], schizophrenia [57], depression [58], anxiety, and sleep disorders (Younis & Abd Alhammid, 2023). This substantial number highlights not only the versatility of nano- and microemulsions as drug delivery systems but also reflects the growing scientific effort to overcome the challenges posed by the blood–brain barrier through intranasal administration. The wide range of treated conditions underscores the importance of intranasal nanotechnology as an emerging therapeutic tool with the potential to reshape the landscape of neurological treatment in the coming years.

The prominence of articles targeting the brain through intranasal nano- and microemulsions is no coincidence. These studies respond to a critical need in medicine: finding effective, non-invasive ways to treat neurological disorders that are notoriously difficult to manage due to the limitations imposed by the blood–brain barrier. The combination of innovative formulation strategies with a route of administration that offers direct access to the CNS has naturally drawn significant academic attention. Moreover, many of these studies were published in a period when nanotechnology was gaining momentum globally, generally in reputable journals with wide circulation, enhancing their visibility and citation potential.

Other relevant terms include “pharmacokinetics” and “biodistribution”, both located in Cluster 2 and presenting moderate PageRank values, indicating growing interest in evaluating the systemic and local performance of intranasally administered nano- and microemulsions. The presence of “curcumin” and “insulin”, bioactive compounds with low oral bioavailability, demonstrates a trend in the literature toward using the intranasal route to deliver drugs with biopharmaceutical challenges, particularly hydrophobic molecules or macromolecules sensitive to enzymatic degradation.

The presence of “Alzheimer’s disease” in Cluster 1 underscores the strategic role of intranasal nanoemulsions within the complex therapeutic landscape of neurodegenerative disorders. This connection reflects a growing alignment between advanced drug delivery technologies and the urgent clinical need to address diseases where conventional approaches have repeatedly fallen short. It speaks to both the scientific ambition of overcoming the formidable challenges of brain drug delivery and the human drive to find effective, less invasive treatments for conditions that profoundly impact quality of life. Ultimately, this convergence of technological innovation and clinical relevance raises a critical question: will the pace of research be matched by the translation of these advances into therapies that patients can truly access, or will these breakthroughs remain largely confined to academic literature? The costs of developing and testing such technology are high, and will likely remain high for patients, but the benefits must ultimately outweigh those of current treatments.

Therefore, the data demonstrate that the field of intranasal nano- and microemulsions is structured around thematic axes involving the administration route, brain-targeting strategies, technological characteristics of the formulations, and clinical applications in neurological disorders. The interconnection among these elements reinforces the relevance of nano- and microemulsions as innovative delivery systems, with the potential to redefine the paradigm of brain drug delivery in a safe, effective, and non-invasive manner.

### 3.7. Most Frequently Used Drugs

The scientometric analysis revealed that among the articles published between 2004 and 2024 on nano- and microemulsions for intranasal application, certain drugs stood out due to their frequency of use and therapeutic relevance. To deepen the understanding of the most promising clinical trends and applications, the five most frequently cited drugs in the literature were selected. Below, the main characteristics of each compound are described, with emphasis on their therapeutic effects and the benefits associated with intranasal administration via nano- and microemulsions, particularly in the treatment of neurodegenerative, psychiatric, and other neurological disorders.

#### 3.7.1. Curcumin

Curcumin was the most frequently used compound among the articles analyzed, appearing in 16 out of 379 studies, either as a single agent or in combination with other drugs. As the primary bioactive component of turmeric, curcumin exhibits antioxidant, anti-inflammatory, and neuroprotective properties, making it a promising therapeutic agent for CNS disorders [59,60]. However, its low water solubility and limited oral bioavailability have driven the development of advanced drug delivery systems, such as nano- and microemulsions for intranasal administration [61,62,63]. This route enables curcumin to reach the brain directly, bypassing the BBB and enhancing its neuroprotective effects [64]. Studies have demonstrated its efficacy in models of Alzheimer’s disease, Parkinson’s disease, and depression, as well as its anxiolytic activity and ability to modulate inflammatory and oxidative pathways involved in the progression of neurodegenerative disorders [59,60,61,63,64,65,66].

The predominance of curcumin as the most frequently encapsulated compound in the analyzed literature likely reflects a convergence of its broad pharmacological potential and the need for innovative therapeutic strategies across multiple disease contexts. Its well-documented effects such as spanning antioxidant [67], anti-inflammatory [65], anticancer [68], antimicrobial [66], and neuroprotective activities [69], render it a candidate for diverse clinical applications, from oncology to neurodegenerative disorders [61,62,65,68]. Moreover, curcumin’s favorable safety profile, natural origin, and long history of dietary use strengthen its translational appeal compared to many synthetic agents. At the same time, its intrinsic biopharmaceutical limitations, including extremely low aqueous solubility, chemical instability, and rapid systemic metabolism, have positioned it as a prime target for encapsulation research, driving the exploration of delivery platforms such as nano- and microemulsions. The adaptability of these systems to various administration routes, including intranasal, oral, and topical, further amplifies their relevance, enabling both localized and systemic therapeutic effects while overcoming key barriers such as the blood–brain barrier. Consequently, curcumin serves not only as a model compound for validating novel nanocarriers but also as a versatile therapeutic scaffold whose delivery could have different implications across a spectrum of chronic and hard-to-treat diseases.

#### 3.7.2. Quercetin

Quercetin was the second most frequently used drug in the analyzed literature, appearing in 6 out of 379 articles involving nano- and microemulsion-based intranasal delivery. Quercetin is a natural flavonoid with well-established antioxidant and anti-inflammatory properties that has gained prominence in intranasal nano- and microemulsion formulations due to its neuroprotective potential [70]. Its application is linked to the treatment of neurodegenerative diseases such as Alzheimer’s and Parkinson’s, through the mitigation of oxidative stress and neuroinflammation [51,70,71,72]. The intranasal route bypasses the BBB, allowing for more efficient brain delivery [73]. Additionally, studies have shown beneficial effects in models of depression and cognitive disorders, reinforcing quercetin’s role as a promising candidate in nanometric delivery strategies targeting the CNS [51,71,72].

The recurrent selection of quercetin in nano- and microemulsion-based intranasal delivery research reflects its unique intersection of pharmacodynamic versatility in the therapeutic needs in CNS disorders. Beyond its well-recognized antioxidant [72] and anti-inflammatory activities [70], quercetin exhibits multi-target modulation of key molecular pathways, including the inhibition of pro-inflammatory cytokines, regulation of mitochondrial function, and attenuation of excitotoxicity [51,70,72], mechanisms relevant to the pathophysiology of neurodegeneration. Its ability to influence both neuronal survival and synaptic plasticity positions it as a compound capable of not only mitigating disease progression but also supporting functional recovery. However, similar to other flavonoids, its clinical translation has been hindered by poor aqueous solubility, low gastrointestinal absorption, and rapid metabolism, making it an ideal candidate for advanced nanocarrier systems. This combination of mechanistic breadth, disease applicability, and delivery feasibility helps explain why quercetin has emerged as a recurring focus in CNS-targeted nano- and microemulsion research, despite the availability of numerous other bioactive flavonoids.

#### 3.7.3. Carbamazepine

Carbamazepine, widely used in the treatment of epilepsy and mood disorders, has been formulated in intranasal nano- and microemulsions to enhance its brain bioavailability and reduce systemic side effects [74]. Among the 379 articles reviewed, 5 utilized carbamazepine in their formulations. This approach is particularly relevant for conditions such as refractory epilepsy and bipolar disorder, where rapid central action is desirable [75,76]. Intranasal administration of nanoencapsulated carbamazepine facilitates a faster onset of therapeutic action and is also being investigated in the context of schizophrenia and sleep disorders due to its modulation of neuronal sodium channels [74,75,76,77].

The amount of carbamazepine in intranasal nano- and microemulsion research can be attributed to its role in managing neurological and psychiatric conditions that demand both precise dosing and rapid therapeutic onset. As a first-line antiepileptic and mood-stabilizing agent, its clinical utility extends beyond seizure control to the modulation of affective and psychotic symptoms, reflecting a broad neuropharmacological spectrum. However, conventional oral administration is often limited by delayed absorption, variable bioavailability, and extensive hepatic metabolism, which can contribute to dose fluctuations and adverse effects [78]. By leveraging nano- and microemulsion-based intranasal delivery, researchers aim to achieve more consistent and predictable brain concentrations, reducing latency to peak effect, an advantage of particular importance in acute seizure management and mood stabilization during crises [77]. Furthermore, the potential to lower systemic exposure while maintaining or enhancing central efficacy offers a compelling rationale for this approach, especially in long-term therapy where cumulative toxicity is a concern.

#### 3.7.4. Diazepam

Diazepam was used by five authors in the preparation of nano- and microemulsions. As a benzodiazepine widely employed for the management of anxiety, seizures, and sleep disorders, it has shown high compatibility with nano- and microemulsion formulations for intranasal use [79,80,81]. This route of administration enhances rapid onset of action and targeted brain delivery, making it ideal for managing seizure crises and panic attacks [81,82,83]. Intranasal diazepam nano- and microemulsions represent an effective alternative for emergency interventions, particularly in patients with swallowing difficulties or compromised venous access. Additionally, they are being explored in treatment protocols for generalized anxiety and insomnia [82,83].

Diazepam’s recurrent inclusion in nano- and microemulsion research is strongly linked to its pharmacological profile, which demands rapid CNS penetration to achieve optimal therapeutic outcomes in acute and high-risk scenarios. As a lipophilic compound with high membrane permeability, diazepam is inherently well suited to nano- and microemulsion systems [84], where the fine droplet size and surfactant-mediated stabilization can further enhance mucosal absorption. In the intranasal context, this translates into swift drug delivery along olfactory and trigeminal pathways, bypassing gastrointestinal absorption and hepatic first-pass metabolism, critical advantages when immediate symptom control is required, such as in-status epilepticus or severe panic episodes.

#### 3.7.5. Insulin

Although insulin does not have a direct target in the brain, studies have shown that the intranasal route offers a promising solution for improving treatment acceptability and discretion. This protein appeared in 5 of the 379 analyzed articles. Insulin remains an essential drug for the management of diabetes mellitus, particularly in patients with absolute or relative hormone deficiency [85]. However, challenges such as rapid enzymatic degradation, the need for multiple daily administrations, and fluctuations in plasma concentration have driven the development of more efficient delivery systems [85,86]. The formulation of insulin into nano- and microemulsions for intranasal administration has emerged as a promising strategy. This approach enables controlled drug release, faster absorption, and a less invasive alternative that enhances patient adherence. Intranasal delivery also offers additional advantages, such as reduced discomfort compared to subcutaneous injections and the potential to bypass hepatic metabolism, favoring a more rapid and direct effect on glycemic control [87,88,89,90,91]. Therefore, intranasal insulin nano- and microemulsions represent a significant advancement in diabetes therapy by combining efficacy, convenience, and improved patient comfort.

Over the two decades analyzed, a clear thematic evolution in intranasal nano- and microemulsion research has emerged, marked by a gradual shift from predominantly synthetic active pharmaceutical ingredients to natural bioactive compounds. Initial studies favored synthetic molecules, often repurposed from existing CNS therapeutics, reflecting their well-characterized pharmacodynamics and established clinical use. However, growing concerns about long-term toxicity, adverse effect profiles, and patient adherence, coupled with growing social and regulatory pressure for safer and more sustainable interventions, have fueled interest in naturally derived compounds such as curcumin, quercetin, and resveratrol. Not only the pharmaceuticals themselves, but also the materials used to produce these nanoproducts. Synthetic polymers such as PLGA, PLA, and PEG have been replaced by natural compounds such as zein, gliadin, chitosan, and others. This is also true in micro- and nano-emulsions; the use of industrially produced oils has given way to natural oils such as corn oil, soybean oil, and others.

This transition is further supported by advances in nano- and microformulation technologies, which address the biopharmaceutical limitations of low solubility, instability, and low bioavailability that have historically hindered the clinical translation of many natural products. This trend suggests a strategic and potentially beneficial evolution in the field, where the convergence of natural product pharmacology and nanotechnology can expand the therapeutic landscape while addressing currently hotly debated environmental demands, such as solvent use, equipment use, and manufacturing materials that generate plastics and investigating other products that can be recycled, contributing to the physical health of patients as well as the health of the planet.

## 4. Conclusions and Future Perspectives

A scientometric study spanning two decades revealed a remarkable advancement in research on nano- and microemulsions for nasal administration, with a significant surge beginning around 2010. This growth reflects the increasing scientific focus on strategies capable of overcoming physiological barriers, such as the BBB, thereby enhancing therapeutic efficacy, enabling targeted drug delivery, and reducing systemic side effects. The combination of the nasal route with nano- and microemulsion-based formulations has emerged as a highly promising, non-invasive, efficient, and well-tolerated strategy in the treatment of neurological and psychiatric disorders.

Among the 379 studies analyzed, a wide range of active pharmaceutical ingredients was identified, with particular emphasis on compounds such as curcumin, quercetin, carbamazepine, diazepam, and insulin. The analysis of the most frequently used drugs highlights the versatility of nano- and microemulsion technology, which supports the delivery of both hydrophilic and lipophilic molecules, encompassing diverse mechanisms of action and therapeutic applications. Furthermore, the keyword co-occurrence analysis reinforced the relevance of nano- and microemulsions in brain-targeted drug delivery, especially in conditions such as Alzheimer’s disease, Parkinson’s disease, brain tumors, schizophrenia, and sleep disorders.

All of these findings point toward important future directions for the field. Non-invasive drug delivery routes, the use of natural products, and increasingly green synthesis pathways represent promising trends, as they not only minimize patient discomfort but also contribute to environmental sustainability. In a world increasingly affected by chemical pollutants, fossil fuel consumption, and excessive water use, such approaches offer a more responsible and patient-centered alternative. Targeted therapies for conditions such as cancer and psychiatric disorders via non-invasive methods may improve treatment adherence and reduce adverse effects, ultimately enhancing patient outcomes.

Research investment must also be emphasized, as scientific advancement depends heavily on adequate funding. While high-income countries continue to lead in research funding, emerging economies such as India have shown growing interest and investment in scientific innovation, India, in fact, ranked first in publication volume in the present study.

Moreover, co-occurrence networks revealed through scientometric analysis highlight the relevance of international collaboration. Such partnerships can lead to more comprehensive studies across diverse populations, serving a shared global interest. In this context, scientometrics emerges as a powerful tool not only for mapping research trends and collaboration patterns, but also for identifying potential strategic partnerships among researchers in the field. Future research is expected to increasingly explore personalized approaches, interdisciplinary collaborations, and clinical validation, consolidating intranasal nano- and microemulsions as a transformative strategy in nanomedicine.

Despite the extensive body of preclinical research on intranasal nano- and microemulsions, clinical applications remain scarce due to a series of well-recognized translational barriers. One of the primary limitations lies in the complex regulatory landscape, which lacks harmonized and specific guidelines for nanocarrier-based intranasal delivery systems. While preclinical data often demonstrate robust efficacy and safety in animal models, the transition to human trials demands strict compliance with quality, safety, and manufacturing standards. In the absence of clear regulatory frameworks in every regulatory agency, such as the FDA, the EMA, and the ANVISA, these requirements can significantly delay or halt clinical advancement.

Another critical obstacle is the challenge of scaling up formulations from laboratory to industrial production. Many systems developed at the bench rely on excipients, processing conditions, or physicochemical properties that compromise long-term stability or fail to meet Good Manufacturing Practice standards. This lack of scalability reduces their feasibility for commercial development and clinical deployment.

Safety considerations also remain a key limiting factor. Most studies focus on acute toxicity and short-term tolerability, with far fewer addressing long-term safety endpoints, including potential mucosal irritation, immunogenicity, bioaccumulation, or chronic effects on nasal epithelium integrity. Without comprehensive toxicological profiles, regulatory approval, clinical adoption becomes inherently more difficult.

Physiological and anatomical variability of the human nasal cavity further complicates translation. Factors such as individual differences in mucociliary clearance rates, nasal airflow patterns, and pre-existing respiratory conditions can significantly influence drug deposition and absorption, limiting the predictive value of preclinical models.

Finally, economic and commercial considerations play a decisive role. The development of advanced nanocarrier formulations involves high manufacturing costs and considerable financial risk, particularly when market advantages over existing, approved therapies remain uncertain. As a result, industry engagement in large-scale clinical trials for intranasal nano- and microemulsions is often limited, slowing the transition from laboratory innovation to patient care.

Based on these findings, the field of nasal nano- and microemulsions is expected to continue expanding, driven by advancements in nanotechnology, pharmaceutical innovation, and materials science. Future directions in intranasal nano- and microemulsion research could prioritize the development of mucoadhesive-enhanced formulations, as these systems can prolong residence time in the nasal cavity, counteracting the rapid clearance associated with mucociliary transport and thereby improving drug absorption and brain-targeting efficiency, while also using “planet-friendly” products. The incorporation of safe and well-characterized permeability enhancers is equally important, as these agents can transiently modulate epithelial tight junctions or facilitate transcellular uptake without causing long-term mucosal damage, addressing one of the key limitations in delivering macromolecules or drugs with poor permeability to the CNS. Furthermore, the integration of pharmacogenomics into formulation design and therapeutic planning represents a step toward personalized medicine, allowing intranasal treatments to be tailored according to individual genetic profiles that influence drug metabolism, receptor expression, and transport mechanisms. By combining these strategies, future research can bridge existing translational gaps, enhance both the efficacy and safety of intranasal delivery systems while aligning with regulatory expectations for patient-centered drug development with quality and accessibility.

It is important to acknowledge that this study relied exclusively on Scopus as the data source, a choice made for technical and practical reasons that ensured consistency in data extraction and analysis. While this approach did not capture approximately 34% of unique records indexed only in Web of Science (WoS), it still provided a comprehensive and representative overview of the field. Nevertheless, future investigations may benefit from integrating multiple databases to further expand coverage and reinforce the robustness of scientometric analyses.

Strengthening interdisciplinary collaborations and establishing clinical protocols will be essential to validate the safety and efficacy of these systems in humans. The synergy between basic science, technological innovation, and supportive research policies will be key to translating the potential of nasal nano- and microemulsions into effective solutions for complex and globally impactful diseases.

## Figures and Tables

**Table 1:** Search strategy from the Scopus core collection.

Research Database	Scopus Core Collection
Citation indexes	CiteScore, SJR, SNIP, Field-weighted Citation Impact and PlumX Metrics
Search query formulation	Search: (Nanoemulsion * OR Microemulsion *) AND (Nose-to-brain OR Intranasal)
Documents types included	Complete research articles
Documents types excluded	Reviews, Book Chapters, Retractions, Letters to the Editor, Conference articles, Erratum, Editorial, Short Survey and Abstracts
Search period	From 1 January 2004 to 31 December 2024
Data collection	Export with full records and cited references in plain text, bib and csv format
Duplicate documents	0 duplicate.
Sample size (included and excluded)	A total of 614 records were retrieved; after applying the time filter, 582 remained. Of these, 379 research articles were included, while 165 reviews, 20 book chapters, 10 conference articles, 3 short surveys, 2 errata, 2 editorials, and 1 retracted paper were excluded.

* acts as a Boolean operator to capture singular and plural forms of keywords.

**Table 2:** The ten most-cited journals and those with the highest number of publications between 2004 and 2024.

Sources	Articles	TC	H-Index
*International Journal of Pharmaceutics*	27	1783	18
*Journal Of Drug Delivery Science and Technology*	18	513	14
*AAPS PharmSciTech*	14	492	10
*Drug Delivery*	14	946	14
*Pharmaceutics*	14	442	9
*Molecular Pharmaceutics*	9	245	8
*Vaccine*	8	216	7
*Artificial Cells, Nanomedicine and Biotechnology*	7	272	7
*Current Drug Delivery*	7	204	6
*Drug Development and Industrial Pharmacy*	6	248	6

TC: total citations. Source: Bibliometrix through Scopus data, 2025.

**Table 3:** Most-cited articles on the topic of intranasal nano- and microemulsions between 2004 and 2024, with methodological and technological innovations.

Reference (Journal, Year)—TC/TCPY	Core Methodological/Technological Innovation	Platform/Formulation Specifics	Model and Evaluation Approach	Practical Implications for Intranasal Nano-/Microemulsions
Dhuria et al. [45] (*Journal of Pharmaceutical Sciences*, 2010)—1043/65.19	Integrates anatomical/physiological pathways (olfactory, trigeminal, vasculature, CSF, lymphatic) with operational variables (pH, osmolarity, mucoadhesives/permeation enhancers, administered volume, head position, application technique).	Route-level design space rather than a specific formulation.	Synthesis of experimental evidence and practice parameters.	Provides a design checklist for intranasal systems: optimize formulation attributes and administration technique to enhance CNS targeting while minimizing systemic exposure/adverse effects.
Kumar et al. [46] (*International Journal of Pharmaceutics*, 2008)—421/23.39	Nanoemulsion vs. mucoadhesive nanoemulsion head-to-head (RNE vs. RMNE); integrates high-energy emulsification, rigorous physicochemical optimization, and in vivo targeting. Radiolabeling (^99^ᵐTc-risperidone) for biodistribution.	RNE and RMNE; drug content 98.86%/99.12%; pH 3.5–6.5; size 15.5–16.7 nm; zeta potential measured. RMNE showed ↑ brain uptake, ↑ DTE%, ↑ DTP% vs. comparators (values not specified in text).	Rats; intranasal vs. intravenous; brain and blood distribution quantified.	Demonstrates mucoadhesion as a tractable lever for direct transport and enhanced CNS delivery in nanoemulsions; template for translational pipelines (formulation → in vivo).
Patel et al. [47] (*CNS Drugs*, 2017)—345/38.33	Consolidates physiological transport tactics (receptor-mediated, efflux inhibition) and nanostructured systems (liposomes, nanoparticles, nanoemulsions, dendrimers), plus alternative routes (intranasal) and transient BBB opening.	Cross-platform synthesis (no single formulation).	Narrative emphasizing 2015–2016 advances.	Positions intranasal nanoemulsions within a broader toolkit for CNS delivery; guides hybrid/combined strategy selection in future studies.
Keller [28] (*Drug Delivery and Translational Research*, 2022)—342/85.50	Centers safety profiling, local nasal toxicity, and regulatory considerations; inventories drugs already approved intranasally; calls for formulation optimization to ensure safety + efficacy.	Route-level risk management; not a single platform.	Critical analysis of development pipeline with safety emphasis.	Embeds toxicology and regulatory compliance into early formulation decisions; essential for clinical translation of nano/microemulsions.
Zhang et al. [48] (*International Journal of Pharmaceutics*, 2004)—294/13.36	Rational o/w microemulsion design for poorly soluble NM; optimized with Labrafil M 1944CS + Cremophor RH 40/ethanol + water; demonstrates nasal safety and CNS targeting.	Solubility 6.4 mg/mL; droplet ~30 nm; non-ionic surfactants. BA 32% (IN); ~3× higher olfactory bulb uptake vs. IV; ↑ brain and CSF distribution; no nasal toxicity observed.	Rats; intranasal vs. intravenous; brain and CSF distribution; olfactory bulb uptake.	Early proof-of-concept that microemulsions can enhance brain delivery and solubility; concrete composition/metrics to emulate in similar lipophilic APIs.

TC: total citations; TCPY: total citation per year; CSF: cerebrospinal fluid; RNE: risperidone nanoemulsion; RMNE: risperidone mucoadhesive nanoemulsion; DTE%: direct transport efficiency; DTP%: direct transport percentage; BA: bioavailability; BBB: blood–brain barrier; API: active pharmaceutical ingredient; ↑: increase. Source: Bibliometrix, thought Scopus data, 2025.

**Table 4:** Centrality metrics of keywords in the co-occurrence network.

Circle	Cluster	Betweenness	Closeness	PageRank
nanoemulsion	4	402.621	0.015	0.128
microemulsion	1	376.261	0.015	0.128
brain targeting	1	162.457	0.014	0.087
intranasal	1	130.565	0.013	0.078
curcumin	2	45.044	0.011	0.022
drug delivery	8	45	0.009	0.012
nose-to-brain delivery	6	45	0.01	0.014
intranasal delivery	1	25.778	0.012	0.047
intranasal administration	2	11.536	0.011	0.028
intranasal drug delivery	1	9.186	0.011	0.021
nasal	1	4.386	0.011	0.012
Alzheimer’s disease	1	4.169	0.011	0.019
insulin	5	3.839	0.011	0.01
chitosan	1	2.52	0.011	0.018
vaccine	3	2.409	0.01	0.026
nanoemulsions	2	2.072	0.009	0.016
mucoadhesion	1	1.631	0.011	0.015
biodistribution	1	1.528	0.011	0.021
brain delivery	2	1.126	0.01	0.015
pharmacokinetics	2	1.032	0.01	0.016

## Data Availability

The data used in this study were obtained from the Scopus database and processed using the Bibliometrix package (version 4.1.3) in R Studio (version 4.3.3) and VOSviewer (version 1.6.20) software for bibliometric analysis and visualization. Due to Scopus licensing agreements and copyright terms, the raw data used in this study can be shared publicly, as long as the source database is properly cited. However, all relevant metadata generated and analyzed during the current study are available from the corresponding author upon reasonable request. The data supporting the findings will also be presented in summary form within this published article.

## Supplementary Materials

The following supporting information can be downloaded at: https://www.mdpi.com/article/10.3390/pharmaceutics17091104/s1, Biblioshiny_Report; Excel Revisões SCOPUS Intranasal; Excel SCOPUS Intranasal; R Script to biblioshine.
